# Retraction

**DOI:** 10.1002/cam4.5771

**Published:** 2023-04-25

## Abstract

Wei, Y, Wei, L, Li, J, et al. SLCO4A1‐AS1 promotes cell growth and induces resistance in lung adenocarcinoma by modulating miR‐4701‐5p/NFE2L1 axis to activate WNT pathway. Cancer Med. 2020;9:7205‐7217. https://doi.org/10.1002/cam4.3270

The above article, published online in Cancer Medicine on 6th August 2020 in Wiley Online Library (wileyonlinelibrary.com), has been retracted by agreement between the authors, the journal Editor‐in‐Chief, Dr Stephen Tait and John Wiley and Sons Ltd. The retraction has been requested by the Authors as the study design provided for insufficient results to support the argument, invalidating the conclusions of the paper.

Wei, Y, Wei, L, Li, J, et al. SLCO4A1‐AS1 promotes cell growth and induces resistance in lung adenocarcinoma by modulating miR‐4701‐5p/NFE2L1 axis to activate WNT pathway. Cancer Med. 2020;9:7205‐7217. https://doi.org/10.1002/cam4.3270

The above article, published online in Cancer Medicine on 6th August 2020 in Wiley Online Library (wileyonlinelibrary.com), has been retracted by agreement between the authors, the journal Editor‐in‐Chief, Dr Stephen Tait and John Wiley and Sons Ltd. The retraction has been requested by the Authors as the study design provided for insufficient results to support the argument, invalidating the conclusions of the paper.

